# Development of Planar Illumination Strategies for Solving Mysteries in the Sub-Cellular Realm

**DOI:** 10.3390/ijms23031643

**Published:** 2022-01-31

**Authors:** Tanveer Teranikar, Jessica Lim, Toluwani Ijaseun, Juhyun Lee

**Affiliations:** Department of Bioengineering, University of Texas at Arlington, Arlington, TX 75022, USA; tanveerashwini.teranikar@mavs.uta.edu (T.T.); jessica.lim@mavs.uta.edu (J.L.); toluwani.ijaseun@mavs.uta.edu (T.I.)

**Keywords:** light sheet microscope, super-resolution, axially swept light sheet, oblique plane illumination, lattice light sheet, sub-voxel resolving technique, single-molecule localization light sheet

## Abstract

Optical microscopy has vastly expanded the frontiers of structural and functional biology, due to the non-invasive probing of dynamic volumes in vivo. However, traditional widefield microscopy illuminating the entire field of view (FOV) is adversely affected by out-of-focus light scatter. Consequently, standard upright or inverted microscopes are inept in sampling diffraction-limited volumes smaller than the optical system’s point spread function (PSF). Over the last few decades, several planar and structured (sinusoidal) illumination modalities have offered unprecedented access to sub-cellular organelles and 4D (3D + time) image acquisition. Furthermore, these optical sectioning systems remain unaffected by the size of biological samples, providing high signal-to-noise (SNR) ratios for objective lenses (OLs) with long working distances (WDs). This review aims to guide biologists regarding planar illumination strategies, capable of harnessing sub-micron spatial resolution with a millimeter depth of penetration.

## 1. Introduction 

Optical tomography has contributed tremendously to facilitating the non-invasive, quantitative modeling of tissue homeostasis and biochemical/mechanical mechanisms [1,2,3,4,5,6,7]. Furthermore, the ability to fluorescently label tissue in/ex vivo with minimal perturbation and high specificity [8,9,10,11,12,13,14,15,16,17,18,19,20], has opened doors to hitherto-unidentified cellular mechanisms. As a result, the biomedical research community has been able to characterize aberrant tissue and assimilate developmental biomarkers with high fidelity [21,22,23,24,25,26,27,28,29].

Traditionally, cellular mechanisms are identified in micron thick-tissue sections, using fluorescent dyes, antibodies or genetically encoded proteins [20,26,30,31,32,33,34,35,36,37,38]. However, histological findings are often predisposed to poor inter-/ intra-observer repeatability [37,39,40]. This is attributed to variations in section thickness, blade tilt angle, refractive index mismatch (RIM), and improper tissue handling and fixation protocols, as well as contaminants [41,42,43,44,45,46,47,48]. In particular, the degree of invasiveness involved in physical sectioning inhibits the orchestration of dynamic organogenesis. To this extent, fluorescence imaging has fulfilled the innate desire of biologists to probe anatomical abnormalities or metabolic dysregulation in vivo [2,8,35,49,50,51]

Conventional microscopes illuminating whole biological samples are prone to deleterious background noise from imaging planes beyond the plane of focus [52,53]. As a result, nanoscale organelles, proteins or molecular units in polymers spaced closer than 250–300 nm laterally (λ/2NA) and 550 nm axially cannot be ordinarily resolved [54,55,56,57,58]. The last few decades have witnessed a metamorphosis of whole-slide imaging (WSI) into user-modulated, optical sectioning [8,9,50,52,59,60,61,62] and patterned excitation strategies [52,55,63]. Periodic illumination modalities such as structured illumination microscopy (SIM) [64,65,66], stimulated emission depletion microscopy (STED) [67,68], photoactivation localization microscopy (PALM) [69] and stochastic optical reconstruction microscopy (STORM) [70,71,72] have been developed to overcome the coveted Abbe diffraction barrier [9,50]. This is achieved through the modulation of higher optical frequencies into the visible frequency passband, through periodic, interference illumination patterns [52]. However, the abovementioned super resolution methods are derived primarily from widefield, two-photon or total internal reflection (TIRF) illumination strategies [36,55]. Hence, potential limitations include out-of-focus fluorescence and anisotropic nanoscale resolution for thick biological specimens/large fields of view (FOV) [55,73,74,75]. Moreover, a tradeoff is observed between spatio-temporal resolution, fluorescence labeling density and laser power required to avoid photo bleaching for a millimeter depth of penetration [56,59,73,76,77,78,79,80]. In this regard, planar illumination has successfully filled the niche for dynamic, subcellular fluorescence tomography.

Light sheet microscopy (LSM) has emerged as a very attractive optical technique due to abilities such as the reconstruction of 4D (3D + time) organogenesis several millimeters deep inside tissue and isotropic nanometer resolution [11,79,81,82]. The non-ionizing nature of LSM modality enables non-invasive, genotypic and phenotypic biomarker screening, for vertebrate animal models and cell studies alike [8,80]. Consequently, LSM has been used to lift the veil on nuclear and cytosolic molecular assemblies such as the endoplasmic reticulum (ER) or mitochondria [13]. Furthermore, LSM has been used to visualize complex neuronal processes or dendritic spine pathophysiology in situ. Notably, such subcellular processes are routinely subject to noise and/or poor framerates for confocal laser scanning and widefield microscopy [11,12,14]. This review aims to cover recently developed planar illumination strategies that have enabled the rapid acquisition of subcellular volumes, hidden behind the optical barrier.

## 2. Light Sheet Modality Considerations

**Figure 1:**
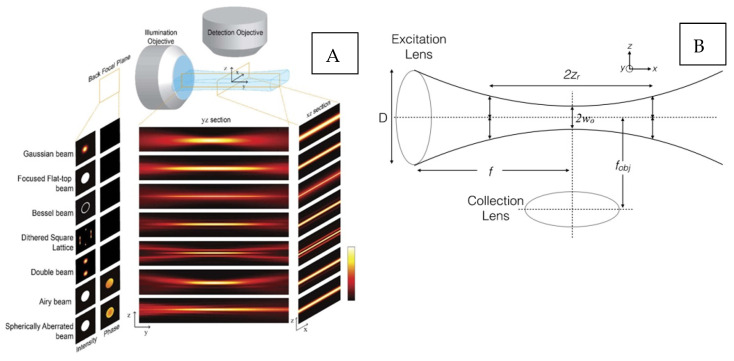
LSM beam profile characteristics. (**A**) LSM single lobe and multilobe top-view and side-view intensity distributions [84]. (**B**) Gaussian light sheet hyperbolic beam profile, D = lens diameter, Zr = Rayleigh length, w0 = beam waist, f = focal length of objective lens [83].

Light sheet fluorescence microscopy (LSM) utilizes hyperbolic optical sections with a Gaussian intensity distribution, for non-invasive sectioning (Figure 1B) [52,60,83,84,85,86]. Consequently, the collection objective lens (OL) (Figure 1B) acquires widefield, static (2D) images in the orthogonal perspective with respect to the illumination OL. In contrast, confocal modalities involve laterally scanned optical sections using a beam with an infinitesimal diameter [52,53]. Volumetric reconstruction typically requires sample translation through a stationary light sheet, with the help of stepper motors, piezo actuators, etc. [60]. Conversely, fixed samples are scanned rapidly by laterally displaced light sheets, analogous to the confocal line-scanning mechanism mentioned previously. The Gaussian light sheet is generated by inserting a mechanical slit in the optical train, to modulate the beam numerical aperture (NA) and the light sheet’s thickness [51,60,62]. On the other hand, a cylindrical lens is used to focus the Gaussian beam onto the illumination OL back aperture [14,86,87]. Improvements to conventional LSM optical geometry include dual excitation (dLSM), for achieving isotropic contrast [60]. Excitation from two orthogonal perspectives reduces stripe artifacts observed by light propagation through heterogenous tissue refractive indices. Multi-view dLSM has gained popularity for isotropic volumetric resolution, allowing multiple imaging perspectives to be acquired via the angular rotation of the sample [60,88].

In this review paper, we discuss ‘single-lobe’ light sheets obtained from Gaussian beams, alongside more complex, ‘multiple-lobe’ light sheets (Figure 1A). Single-lobe planar microscopy results in a monotonically reducing intensity distribution, following the Gaussian peak [84]. On the contrary, multi-lobe planar microscopy contains secondary intensity distributions following a main Gaussian peak. Such ‘side-lobes’ do not monotonically reduce and contain significant magnitude, as compared to Gaussian ‘side-lobes’ [84]. Hence, a lateral FOV for a collection OL can be described in terms of the confocal region, b = 2 ∗ Zr $=2\pi {\left(wo\right)}^{2}/\lambda$, where Zr = Rayleigh length, λ = wavelength and w0 is the beam waist [52,53,83]. The confocal region signifies near-homogenous illumination. Axial resolution can be defined as Raxial = 2wo = 2 nλ/2π NA, thereby relating light sheet thickness to sectioning capability [52,53,83]. Increasing the FOV requires thicker light sheets; however, this reduces the axial resolution due to the low NA. For more complex light sheets, only the main Gaussian peak full width half maximum (FWHM) is usually considered [84].

Most importantly, the LSM modality requires refractive index matching (RIM) between the sample, embedding medium and holder configuration, to increase photon collection efficiency (contrast) by camera pixels. Photon propagation through endogenous pigments, proteins, lipids, the cytoplasm and other molecules within tissue is inherently not conducive for RIM [60,89,90]. Consequently, tissue clearing has proven to be highly efficacious for reducing light absorption/scattering effects associated with widefield detection modalities and improving the depth of penetration. In comparison, optical sectioning through pinhole-based detection is highly robust against background fluorescence [60]. Over the last decade, several tissue-clearing methods, such as PEGASOS, CUBIC, CLARITY, uDISCO, vDISCO and CHAPS/SHANEL, have been developed to achieve sample transparency and reduce RI variability [89]. Organ transparency is achieved through bio-chemical interactions such as the disassociation of collagen, delipidation, decalcification, dehydration and decolorization [89]. Tissue clearing is highly beneficial for high throughput, non-invasive, genotypic or phenotypic mapping in toto [90].

Furthermore, several post-acquisition image processing techniques have been made accessible to microscopists in open source software platforms such as MATLAB and ImageJ (also known as Fiji). Image deblurring is performed via deconvolution using an optical system point spread function (PSF), obtained from imaging diffraction-limited beads embedded in agar- or resin-based embedding media [85,91]. Isotropic image contrast across an attenuated FOV is restored using intensity thresholding techniques such as histogram equalization or nonlinear haze removal [60]. Furthermore, median/Gaussian filtering, background subtraction or morphological operations such as image opening and dilation have been effectively used for noise removal [60].

## 3. Axially Sweeping Light Sheet Microscopy

LSM is a non – invasive tomographic technique that allows acquisition of optical cross-sections, by confining fluorescence within 2D planar sheets in the sample [8]. LSM is routinely used to perform in situ/in vivo analysis of cellular structures or dynamic chemical processes, several millimeters deep inside opaque tissue [11,87]. However, LSM faces fundamental constraints imposed by the OL’s numerical aperture (NA) and working distance (WD) [11,87]. 

Biologists/microscopists often face a dilemma in choosing the acquisition/detection OL’s NA according to the object’s size and WD. A high NA drastically reduces the effective WD of an optical system, restricting the light sheet penetration depth, albeit with increase in the spatial resolution due to the capturing of more light rays. On the other hand, low-NA OLs produce thicker light sheets with longer WDs, compromising the signal-to-noise ratio (SNR) for sub-cellular objects of interest [84]. As a result, a puzzling conundrum of choosing the appropriate NA without sacrificing spatiotemporal resolution is often encountered [11]. Using high-NA excitation optics improves axial resolution by producing thinner light sections. However, high NA tends to reduce the confocal parameter of the light sheet. Hence, imaging vertebrate animal models, such as mice, requiring a large WD benefits from low excitation NA OLs. On the other hand, high-NA detection optics are accompanied by a loss in effective WD for thicker specimens, apart from being costly.

**Figure 2:**
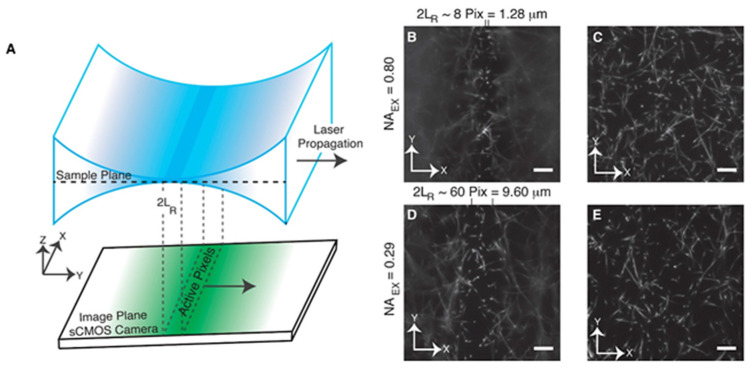
Illustration of ASLSM. (**A**) Schematic of ASLSM. Sample is illuminated with a laser from the y-direction and the fluorescence is taken perpendicular from a camera. L_R_ is the Rayleigh length of the beam. The axial position of the beam is swept in the y-direction with a 2D array of pixels to create an FOV image. (**B**) Image of fluorescently labeled collagen with NA = 0.8. (**C**) Image (**B**) with high optical sectioning. (**D**) Image of fluorescently labeled collagen with NA = 0.29. (**E**) Image of (**D**) with high optical sectioning. (**B**–**E**) Scale bars are 10 µm [87].

To combat this, axially sweeping LSM (ASLSM) **(**Figure 2A) is emerging as a promising optical sectioning strategy for modulating the confocal parameter without sacrificing optical performance. ASLSM optical geometry comprises orthogonal illumination and detection geometry, similarly to conventional LSM [8,11,87]. The modification consists of aberration-free remote focusing mechanisms, displacing the light sheet axially along the optical axis without moving the excitation OL WD. Hence, this enables the the arbitrary tuning of the usable field of view (FOV). The axially sweeping Gaussian light sheet is focused on the back aperture of the excitation OL using a cylindrical lens, akin to conventional LSM light sheet generation, as described in previous sections.

Early manifestations of ASLSM optical geometry, proposed by Dean et al. [87], consisted of averaging axial displacements of the light sheet with the camera’s framerate. The light sheet refocus mechanism across the excitation FOV was realized through wavefront shifts produced by a piezo actuated mirror, through a secondary remote refocus excitation OL [11,87]. The authors reported a sub-micron lateral resolution of 380 ± 20 nm, matched by an axial resolution of 390 ± 6 nm for a high-NA (0.8) excitation OL. Using a low excitation OL NA (0.29) did not result in a lateral resolution loss, although it showed a slight reduction in axial resolution to 919 ± 8 nm [87] due to the reduced aperture of the optical system (Figure 2B–E). The authors comprehensively described the utility of achieving an isotropic 390 nm optical resolution across large confocal ranges (~10 µm) by studying the morphological and functional aspects of individual molecules during organogenesis [84]. ASLSM was able to visualize the anisotropic morphology of independent MV3 melanoma cells labeled with *Tractin-eGFP*, embedded in a collagen volume spanning 162 µm × 162 µm × 100 µm [11,87]. Moreover, ASLSM was capable of visualizing transient phenotypes of vimentin filaments, microtubules and mitotic spindles during epithelial-mesenchymal transition (EMT) [11,87].

Dean et al. further proposed a virtual-scanning ASLSM variant called diagonally scanned LSM (DSLSM) to improve camera framerates by avoiding the physical sample translation required for optical sectioning [12]. Digital light sheet scanning of a conventional 2D cell culture at 45 degrees with respect to the optical axis enabled impressive framerates (3.1Hz) [12]. As a result, DSLSM was able to capture actin and microtubule dynamics, clathrin-mediated endocytosis (CME) and phosphoinositide signaling [12]. Moreover, DSLSM was able to achieve a lateral resolution of 379 ± 18 nm and an axial resolution of 725 ± 29 nm, using a dithered Gaussian lattice illumination mode for homogeneity [12].

Chakraborty et al. [11] further sought to improve previous ASLSM optical performance with the use of multi-immersion excitation OLs, and reported a landmark axial resolution of 260–290 nm using a high-NA (0.7) excitation OL [11,92]. The authors demonstrated the depth penetration ability of ASLSM via the volumetric reconstruction of neurons and dendritic spines, 2.5 mm deep in a PEGASOS-cleared *Thy1-GFP* mouse brain [11,92]. Furthermore, they highlighted the significance of achieving isotropic performance using a low-excitation NA OL by reconstructing individual neonatal kidney cells in a 3.4 × 2.6 × 2.5 mm volume. Other imaging highlights include the visualization of human stem cells (HSCs), nerve fibers and vasculature in PEGASOS-cleared mouse bone marrow at an isotropic 300 nm resolution using a high-NA (0.7) detection OL [11].

However, the axial displacement of the LS beam waist using mechanical remote focusing in previous ASLSM approaches is unable to match or exceed camera readout speed [11,87,93]. Hence, the physical motion involved in voice coil actuators, piezo motors and galvo mirrors requires the time averaging of displaced light sheet foci across the FOV [93]. Landry et al. demonstrated the use of linear phased arrays to achieve superb camera gating times of 350 kHz, enabling faster axial light sheet movement (2.85 µs) compared to the camera pixel refresh rate (9.6 µs). A ‘rolling’ shutter detection scheme was implemented via the modulation of the offset in activating camera pixels across the field of view (FOV) in synchronization with the moving light sheet focus. This enabled the authors to achieve a sub-micron lateral resolution of 504 ± 17 nm, and an axial resolution up to 720 ± 55 nm–889 ± 65 nm [93]. The novel axial scan setup was able to successfully capture 8- and 14-hour-old drosophila embryogenesis at impressive frame update times up to 9.6 µs.

The aforementioned ASLSM techniques have expanded the scope of nanoscale microscopy, which is often limited to low WD OLs. The integration of ASLSM with tissue clearing or tissue expansion methods can be used to perform tracking, analyze morphological changes or quantify chemical concentrations of individual molecules several millimeters deep in large biological volumes with high fidelity.

## 4. Oblique Plane Microscopy

Conventional LSM is based on non-collinearity between excitation and detection OL foci. Hence, the modality is fundamentally constrained towards low-NA excitation OLs with long working distances to avoid optical complexity [12,14,87]. Furthermore, in vivo volumetric reconstruction is adversely affected by the inability to capture light sections close to the coverslip, due to the tradeoff between high-NA detection OLs and WD [14]. High-NA lenses with small WDs significantly improve spatial resolution and single-molecule localization. However, potential drawbacks include the fabrication of custom cuvettes or immersion chambers, also affecting imaging sterility [14]. With respect to these issues, oblique plane microscopy (OPM) has been making tremendous strides in user-accessible, in situ/in vitro single-molecule localization and diffraction-limited imaging. The LSM modality uses the same NA OL for excitation and detection, enabling compatibility with traditional inverted or upright optical geometries. As a result, imaging complexity is greatly reduced due to its compatibility with commercially available/custom fabricated well plates and culture dishes.

An elegant OPM solution for subcellular imaging was proposed by Gustavsson et al. [81,94] in the form of tilted LSM with 3D PSF (TILT3D) based on the integration of point spread function (PSF) engineering optics in the conventional LSM geometry. The authors propounded an unconventional approach by encoding the axial positions of irradiating sub-cellular objects in the structure of a PSF, and not the confocal parameter of a light sheet. The authors reported the use of double helix PSF (DH-PSF) for nanoscale imaging of a HeLa nuclear lamina (~101–113 nm thick) and the mitochondrial membrane structure [81,94]. Furthermore, TILT3D provided localization accuracy of 3 nm in the XY direction and 7 nm in the XZ direction at 3.3 µm above the coverslip, using a long-axial-range (6 µm) tetrapod PSF [94].

**Figure 3:**
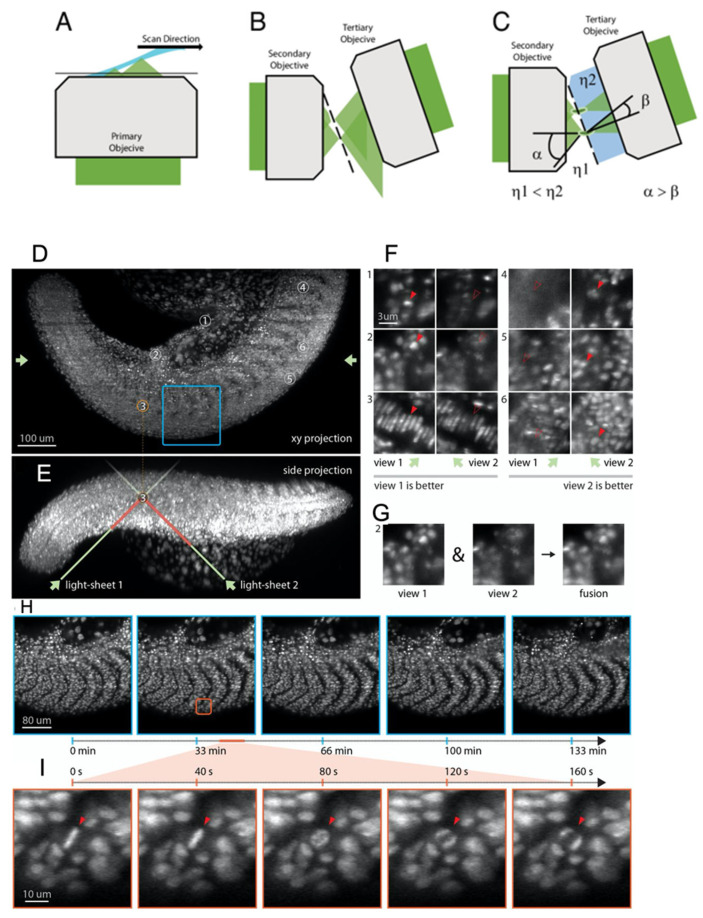
Two illustrations of OPM. (**A**) The light sheet (blue) is from the primary objective lens illuminated at an oblique angle. (**B**) Traditional OPM—Primary objective lens fluorescence is replicated when secondary objective lens crosses with tertiary objective lens. (**C**) Optimal microscope resolution (from α to β) created when light moves from a low refractive index, η_1_, to a high refractive index, η_2_. (**A**–**C**) [14] (**D**) Zebrafish tail at 24 hours post-fertilization with *mCherry*-labeled histones. (**E**) Side projection of light sheets entering sample at 45 degrees. (**F**) Example regions where (left) View 1 has better image quality, whereas (right) View 2 has better image quality. (**G**) Fusion of View 1 and View 2 with better image quality. (**H**) Time-lapse of dorsomedial tail. (**I**) Spatio-temporal image of a cell division. (**D**–**I**) [95].

Recently, Sapoznik et al. [14] proposed a user-friendly OPM optical geometry (Figure 3A–C) method; the primary (excitation and detection) OL (Figure 3A) is used for aberration-free volume scanning. Furthermore, it is also used for forming an intermediate imaging plane with a secondary OL (Figure 3B). The volumetric image formed at the focus of the secondary OL is imaged on camera pixels by a tertiary OL (Figure 3C) [14], thereby avoiding the de-coupling of excitation and detection imaging planes. A custom OL was placed before the camera to increase the effective NA of the optical geometry, by capturing marginal rays that may escape during the virtual light sheet scan. The single-NA optical strategy reported an astounding lateral resolution of ~203 ± 24 nm to 209 ± 33 nm and an equally impressive axial resolution up to 523 ± 60 nm for a large field of view (FOV) of 180 µm × 180 µm, after 20 iterations of Richardson–Lucy deconvolution [14]. The novel OPM technique was used to capture sub-diffraction-limited organelles such as the endoplasmic reticulum in osteosarcoma U2OS cells, vimentin in retinal pigment epithelial cells or the nuclear constriction/herniation mechanisms of melanoma cells in micropore (~2–2.5 µm) environments [14]. Moreover, light sheet virtual scan, as opposed to physical sample translation, enabled high camera framerates (~800 fps) [14]. As a result, the novel OPM method was able to capture calcium translocation due to rat cardiomyocyte contraction assess cytosolic rheological properties and perform the optogenetic activation of Rac1 [14]. The ability to image centimeter-thick tissue without additional image perspectives and integration with traditional coverslip mounting schemes surpasses the native performance metrics of OPM methodologies.

An alternative approach to the single-OL OPM approach proposed by Sapoznik et al. [14] was recently put forth by Yang et al. [95] through the multi-view SR OPM configuration. The authors reported the ability to capture large imaging volumes up to 3000 µm × 800 µm × 300 µm, at a noteworthy resolution of ~450 nm laterally and 2 µm axially [95]. The epi-illumination, DaXi OPM optical geometry was used to capture somatogenesis, along with zebrafish larval embryogenesis and tail development [95] (Figure 3D–I). Furthermore, the ability to switch from upright to inverted illumination allowed simultaneous time-lapse microscopy of nine zebrafish embryos in a glass-bottom Petri dish [95].

Kim et al. [13] further developed an OPM method (obSTORM) capable of delivering a light sheet tunable between 45–90 degree tilt angles. obSTORM reported a resolution of ~44–51 nm for tissue depths of 32 µm, for 45 degree tilt angles. In addition, the authors reported that obSTORM was able to provide an isotropic localization accuracy of ~18.5 nm, hence resolving microtubules (~69 nm) spaced 128 nm apart in fixed A549 cells, along with the mitochondria [13]. The effective FOV of OPM depends on the beam offset at the OL back aperture and the resultant light sheet tilt angle [96]. Although geometrical considerations for OPM FOV are outside scope of this review, the optimization of tilt angles according to sample thickness has been put forth by Kumar et al. [96].

The above-mentioned SR OPM methods have great potential to perform the dynamic imaging of targeted samples or the imaging of whole mounted samples. The sub 200 nm resolution far exceeds traditional widefield or epi-illumination optical limits, ushering in new paradigms for disease modeling in tissue pathophysiology.

## 5. Lattice Light Sheet Microscopy

**Figure 4:**
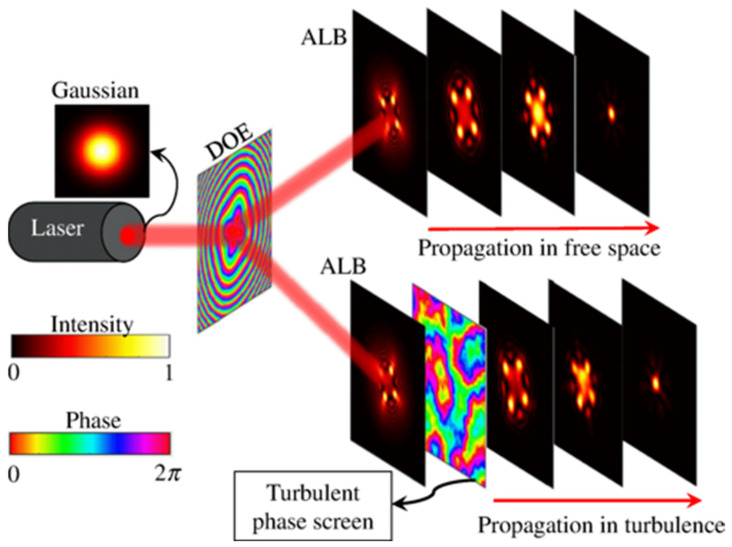
Illustration of how aberration laser beams (ALBs) were produced and propagated in free space and turbulence after passing through an incoming excitation Gaussian beam into a diffractive optical element (DOE) [77].

LLSM has enabled biologists to probe biological volumes across different scales in vivo, due to its excellent optical sectioning (<5 µm), minimal phototoxicity, intrinsic autofluorescence rejection and sub-second imaging rates [78,79]. The LLSM modality produces concentric beam distributions through constructive and destructive interference (Figure 4). However, LLSM is fundamentally constrained by the lens NA, limiting the isotropic nanometer resolution to several hundred microns at best, owing to Gaussian beam waist divergence beyond the Rayleigh range [79]. 

Chen et al. [78] developed sub-micron lattice light sheet microscopy to overcome traditional light sheet drawbacks such as the Gaussian beam scattering tendency and optical section thickness. LLSM relies on extremely thin (0.4–1 µm) optical sections [78,97], created via the modulation of phase masks on the OL aperture that propagate indefinitely without undergoing a change in the beam cross sections [61,88]. The authors reported impressive lateral and axial resolutions of 230 nm and ~370 nm, respectively, for a dithered optical lattice, and a remarkable 150 nm × 280 nm axial *xz* resolution for SIM mode (xz axis) [78]. Furthermore, the authors emphasized the ‘self-healing’ nature of the lattice excitation by describing an astonishing beam width (full width half maximum) of ~1 µm for a propagation distance of 50 µm [78]. Practically, LLSM light sheet propagation length homogeneity depends on modulating the thickness of a ring annulus (ring mask). This is analogous to varying the mechanical/camera pixel rolling shutter slit width in traditional LSM microscopy [78,79]. The optical lattices are periodic fringe patterns produced by the superposition of Bessel beam side-lobes or spatial light modulation before the ring annulus. The authors reported that they were able to observe unrevealed molecular phenomena such as the diffusion and binding kinetics of TMR-Halo-labeled *Sox2* transcription factors in 35 µm thick spheroids, mitochondria and endoplasmic reticulum. The authors also described the ability to track changes in chromosome morphology during each phase of mitosis for HeLa cells or neutrophil migration through a 3D collagen mesh (~8–12 µm travel distance). They suggested the integration of adaptive optics (AO) for improving refractive index aberrations [98].

**Figure 5:**
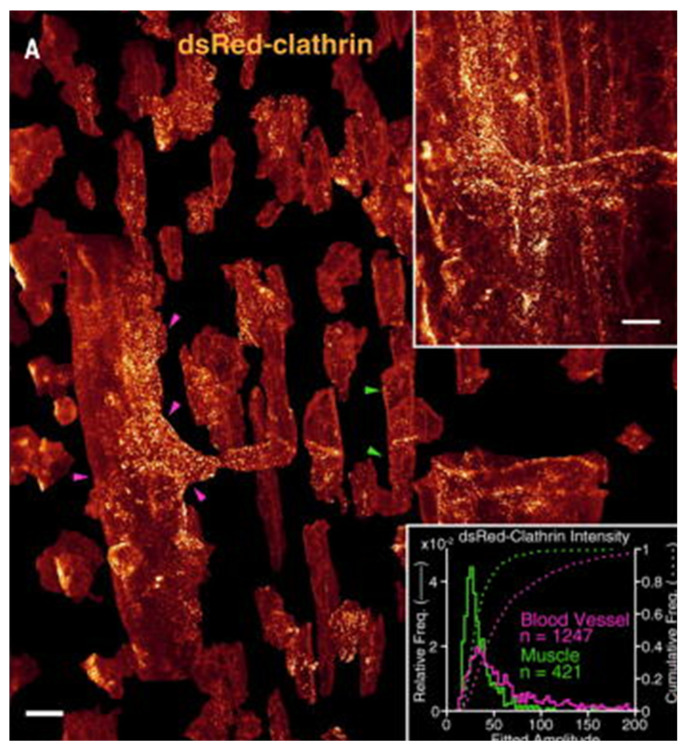
This figure shows separated muscle fibers (examples are shown with green arrowheads) and vascular endothelial cells (examples are shown with magenta arrowheads) that express *DsRed-CLTA*. The upper right corner shows 75 µm by 99 µm by 41 µm of the tail of a developing zebrafish, whereas the bottom right corner shows a graphical depiction of clathrin puncta in the endothelial cells [98]. Scale bars are 10 µm.

Liu et al. highlighted the importance of AO-based LLSM [98], similar to the piezo actuators or galvo mirrors mentioned in previous ASLSM and OPM sections, by visualizing clathrin and dynamin in endocytic pits. They emphasized the lack of poorly defined cell boundaries and the lack of differentiation between membrane structures and cytosol in the absence of aberration-free remote refocus optics [98]. The group proceeded to visualize clathrin-coated pits and vesicles in the dorsal tail region of a zebrafish tail 80 h post-fertilization (Figure 5)**,** along with subcellular organelles such as mitochondria [98]. However, the Bessel mode of illumination for LLSM affects beam attributes such as FOV and beam propagation length. Other potential limitations include the presence of secondary intensity side-lobes (broad side tails), along with a central intensity maximum due to the thickness modulation of the previously-mentioned ring annulus structures [79].

To overcome these limitations, Betzig et al. proposed the integration of a structured illumination mode (SIM) for lattice light sheet microscopy [99]. Three successive phase shifts of discrete, periodic fringes (the fringe separation is less than λ/NA) in a single camera’s exposure period, resulting in a landmark 270 nm full-width at half-maximum (FWHM) [99]. The authors, however, argued that the SIM mode for Bessel-beam-based LLSM was prone to low-SNR image frames due to fringe separation less than λ/2NA. Hence, they sought to improve the contrast of image frames for thicker, multicellular biological samples by means of two-photon illumination (multi-harmonic excitation) [99]. The authors tested the performance of the Bessel-beam-based SIM and two-photon LLSM through a comparison with conventional confocal and digitally scanned LSM. The optical group observed significant phototoxic effects for the confocal imaging of dynamic LLC-PK1 cells, due to the presence of fragmented mitochondria [99]. In contrast, Bessel illumination-based LLSM resulted in non-perturbation of the mitochondrial morphology, along with the preservation of the original intensity (80% photon count) even after the acquisition of 300 biological volumes (individual volumetric stack ~321 images) [99]. Furthermore, the authors reported the ability to track the endoplasmic reticulum (ER) evolution of human osteosarcoma (U2OS) cells, using SIM mode at 300 nm isotropic resolution. Using two-photon excitation, the authors were able to study unrevealed biological phenomena—the retrograde flow of membrane ruffles causing micropinocytosis in an African green monkey kidney cell, the 3D dynamics of chromatid separation during early anaphase and the actin-based filopodia of HeLa cells [99].

More recently, a study was published detailing the integration of LLSM and tissue expansion for whole-brain imaging [100]. The authors reported a 60 nm × 60 nm × 90 nm optical resolution for a 4× tissue expansion. Moreover, they were able to quantify volumes of subcellular organelles, such as mitochondria, and characterize morphological properties for ~1500 dendrites in the mouse brain cortex, as well as the longitudinal myelination of axons [100].

Nanoscale expansion microscopy uses hydrogels to isotropically expand the cellular structure within the specimen, and is capable of resolving structures up to 25–60 nm using conventional optical sectioning techniques [101,102,103]. Single-iteration protocols lead to ~4.5× expansion, whereas multiple iterations (~20) can produce expansion up to 10× [103]. In conjunction with the tissue-clearing techniques mentioned previously, tissue expansion microscopy is a very powerful tool for enabling access to subcellular organelles in millimeter-sized samples, which primarily requires long-WD OLs.

Hence, SR LLSM is emerging as a powerful imaging tool in conjunction with tissue-clearing/expansion methods to learn about the hidden intricacies of biological tissue obscured behind the resolution limit. The independence of the beam intensity profile on lens WD greatly facilitates the nanoscale reconstruction of whole organ systems without a loss in magnification. However, side-lobes in spatial distributions’ of ‘self-healing’ beams such as Bessel and lattices, reduce the optical sectioning resolution. Hence, propagation-invariant beams require post-processing deconvolution, and this warrants further investigation.

## 6. Sub-Voxel-Resolving Light Sheet Microscopy

Sub-voxel-resolving light sheet microscopy (SVR LSM), enables the improvement of spatial resolution through graphics processing unit (GPU)-based computation [82,104], thereby avoiding time-intensive image processing. This modality requires a tilting plate (~10 degrees inclined surface) attached to the sample mount in order to ensure an off-axis scan [82,104,105]. The off-axis scan with the tilting plate enables rapid sub-voxel shifts in both lateral and axial directions [82]. The off-axis shifts are used by the SVR algorithm to reconstruct the final high-resolution output image [82,104,105].

Fei et al. first introduced sub-voxel LSM (SLSM) and revealed that the technique was able to successfully enhance the resolution of a 3D cultured normal human bronchial epithelial (NHBE) cell spheroid [82]. Furthermore, they performed multi-view SLSM and were able to improve the resolution by between four and nine folds, allowing the recovery of human umbilical vein endothelial cell (HUVEC) sprouts. The authors have also reported that SLSM was able to achieve a wide FOV (~233 mm volume), with a low-magnification detection objective lens (NA: 0.1), with a resolution similar to a higher-magnification detection objective lens (NA: 0.45) [82].

**Figure 6:**
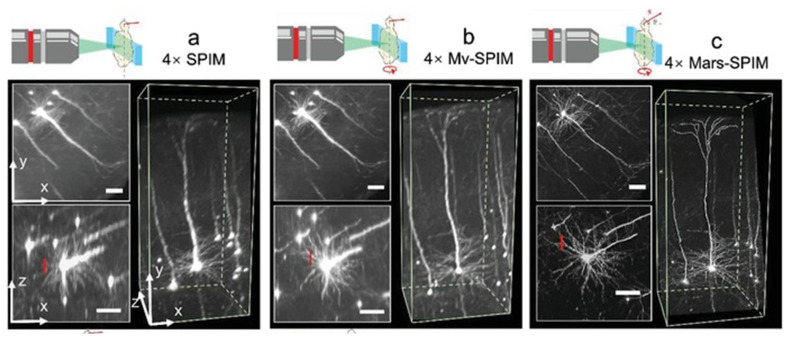
Visualization of neurons on *Tg:thy1-GFP-M* mouse brain. (**a**) 4× imaging on benchtop SPIM with a voxel size of 1.625µm × 1.625µm × 6 µm. (**b**) 4× imaging with multiview SPIM with a voxel size of 1.625µm × 1.625µm × 1.625µm. (**c**) 4× imaging with Mars-SPIM with a reconstructed voxel size of 0.41µm × 0.41µm × 0.41µm [104].

Nie et al. demonstrated an improved resolution (0.975 µm voxel) in a cleared whole mouse brain (*Tg: thy1-GFP-M*), with the implementation of multiangle-resolved sub-voxel selective plane illumination microscopy (Mars-SPIM) [104]. This technique combines the sub-voxel-resolving (SVR) algorithm and the multiview Bayesian deconvolution (MVD) method, bypassing tedious image stitching or interpolation. The continuous capturing of low-resolution images allowed for a total of eight views in whole mouse brain imaging [104]. They also pointed out that the Mars-SPIM technique facilitated the visualization of the clearly resolved neurons in the cortex area by approximately four-fold when compared to conventional SPIM (Figure 6). Thus, this study successfully demonstrated the usage of a computational algorithm to accelerate the performance of a super-resolution microscopy technique without requiring more additional implementation towards optics features.

In another SVR LSM study, conducted by Zhao et al., his group developed a sub-voxel-resolving light-sheet add-on microscopy method (SLAM) that enables light-sheet imaging on conventional fluorescence microscopes [105]. This technique is carried out by designing a portable add-on device that is mounted onto the stage of the inverted microscope [105,106]. The portable device consists of optical components that would have normally been included on a tabletop LSM setup. This setup also includes the customized tilting plate that enables the oblique scanning of the sample, allowing the sub-voxel step shift for super-resolved images. The authors reported the high-throughput mapping of a uDISCO cleared half mouse brain with labeled neurons GFP (*Tg:thy1-GFP-M*) with a super-resolved voxel size of 0.54 µm × 0.54 µm × 3 µm [105]. Furthermore, the SVR algorithm facilitated the resolving of neuronal dendrites that were not clearly visualized in the unprocessed raw images [105]. With its low cost and simple optics advantages, this adaptable add-on device can be utilized on most conventional microscopes for super-resolved images that resemble those obtained using a confocal microscope.

SVR LSMs have been successfully shown to have promising results, surpassing a regular LSM resolution limit by introducing oblique sample tilting and the implementation of a high-throughput reconstruction algorithm flow. The preservation of a large FOV with a subcellular resolution enables the imaging of larger samples that contain detailed fine-structured information that typically cannot be seen. Although most of the alternative SR LSMs require complicated optical geometries, SVR LSM is based on oblique scanning techniques coupled with post-processing algorithms to achieve super-resolved images of larger samples. This effective and inexpensive variant can be added to most existing LSM setups, rendering fast and super-resolved 3D structures in biological specimens.

## 7. Single-Molecule-Localization Light Sheet Microscopy 

A section has been devoted in this review for LSM modalities capable of single-molecule localization via widefield detection. LSM has emerged as a promising candidate for single-molecule localization, owing to minimal photobleaching, the ability to tune penetration depth and the confinement of fluorescence in the imaging plane [107,108]. Furthermore, compatibility with high-NA detection OLs and rapid camera framerates enables excellent spatio-temporal resolution. State-of-the-art modalities such as PALM (photoactivated localization microscopy) and STORM (stochastic optical reconstruction microscopy) often suffer from photo bleaching or poor penetration depth. This is caused by their reliance on total internal reflection (TIRF) or epi-illumination excitation [107,108]. Single-molecule LSM modalities have been used to generate very thin light sections (≤ 7 µm), to localize individual molecules of interest with high fidelity up to several nanometers (lateral localization precision ~30–100 nm, axial localization precision ~135 nm) [107,108,109]. Single-molecule-localization imaging requires a sufficient fluorophore labeling density to minimize axial overlap and high SNR (contrast) to discriminate the background from the foreground.

The first instances of the use of SPIM as a single-molecule-detection strategy was reported by Tokunaga et al. in the form of an oblique light section configuration, namely, highly inclined and laminated microscopy (HILO) [110]. As the name suggests, a light section is inclined via refraction through the coverslip in an inverted/upright fashion, at oblique angles less than the critical angle of TIRF but greater than those in epi-illumination microscopy. The diameter of the light sheet is modulated by controlling the inclination angle of the light sheet, and the authors revealed that light sectioning was achieved up to 7 µm and thinner sectioning was achieved via the insertion of a field stop conjugate to the specimen plane. HILO was used to observe molecular events such as the mediation of nuclear transport after the microinjection of GFP-Importin β into permeabilized cells. A variant of HILO, namely, variable angle epifluorescence microscopy (VAEM), was developed, corresponding to the same time scale, by Konopka et al., and this method is compatible with an inverted microscope or TIRF configuration [111]. Analogously to HILO, VAEM was tested at varying sub-critical angles less than the TIRF critical angle. The authors reported a cohort of novel imaging studies involving cortical microtubules (MT), plasma membrane dynamics in clathrin light chain-GFP (CLC-GFP) and subcellular dynamics concerning the endoplasmic reticulum and Golgi complex in root epidermal cells.

A novel LSM single-molecule strategy was reported by Gebhardt et al. in the form of reflected light sheet microscopy (RSLM), which utilized a polished AFM cantilever arm to decouple the orthogonality of a conventional LSM system [112]. The light sheet was reflected at a 90-degree angle with the help of the cantilever arm, thereby enabling compatibility with traditional coverslip/cell culture sample mounting and high-NA lenses. The authors reported an astonishing light sheet thickness of 1 µm, and the optical system presents exciting prospects for achieving thinner sections (<0.5 µm) with the aid of higher-NA illumination and detection OLs. RSLM was evaluated with respect to HILO for visualizing single-molecule events, such as mEos2-H4 molecules in MCF-7 cell or visualizing nuclear glucocorticoid receptor binding affinity with estrogen receptors. RSLM modality was reported to achieve a 40% improvement in SNR at imaging depths up to 30 µm and a twofold improvement beyond 11 µm, with respect to HILO.

A single-molecule-localization variant of RSLM was developed by Hu et al., substituting the AFM cantilever with a Pellin Broca prism, to decouple the conventional LSM orthogonality [113]. The light sheet was refracted through the prism from the excitation OL onto the imaging sample, at an angle of ~120 degrees with respect to the detection optical train. The authors reported a light sheet thickness of ~1.8 µm and were able to image heterochromatin protein HP1-α in the nuclei of human embryonic stem cells (HESCs).

Furthermore, the integration of varying beam intensity distributions, such as Bessel beams with selective plane illumination, can be used to perform single-molecule localization with relative ease.

Lu et al. reported a Bessel LSM method for ultrathin light sheet confinement, resulting in a lateral full width half maximum (FWHM) of 0.54 µm and an axial FWHM of 1.36 µm, respectively [108]. The Bessel beam intensity distribution was created via the insertion of an annular phase mask in the selective plane illumination optical train. A cylindrical lens was used to produce a one-dimensional collimated Bessel light sheet, i.e., it was used to produce optical astigmatism to prevent fluorophore interference from adjacent imaging planes. The Bessel LSM illumination was reported to resolve structures up to 100 nm; hence, the authors were able to perform volumetric analyses of microtubules in mouse MC3T3-E1 cells and of hippocampal neurons from rat pup brains, as well as of nuclear pore complex proteins (POM121 and Nup153).

Hence, LSM is rapidly developing as a single-molecule-localization technique due to its inherently high SNR and high penetration depth. The modality is empowering biologists to understand nanoscale mechanisms with a high degree of customization, that was previously restricted in the academic environment.

## 8. Conclusions

SR LSM offers monumental advantages over aberration-prone confocal and widefield microscopy, such as rapid optical sectioning and the ability to sample volumetric biological events in vivo or ex vivo with very high spatio-temporal resolutions. Combined with tissue-clearing or expansion methods, LSM allows for the opportunity to investigate molecular structure and function across different scales that were previously unable to be reconstructed without aberrations induced by tissue. In addition, volumetric imaging over time of LSM allows for the dynamic sampling of morphogenesis in various animal models, unaffected by predicaments such as the working distance of the imaging plane or the magnification required by biological applications. Combining LSM with recently developed super-resolution imaging techniques allows the visualization of biomolecular structures in nanometer-scale resolutions, thereby surpassing the diffraction limit in fluorescence microscopy, with higher accuracy. The integration of Bessel beams and Gaussian beams has proven to successfully assist in resolving molecular elements that cannot be resolved at diffraction-limited resolutions. In summary, SR LSM overcomes several pivotal microscopy drawbacks. ASLSM allows for a longer penetration depth without an NA tradeoff; OPM provides an epi-illumination mounting strategy for LSM, enabling better access to conventional cell cultures; SR LLSM offers unparalleled spatio-temporal resolution; and SVR LSM allows for fast, high-throughput volumetric imaging of cleared whole organs at subcellular levels of resolution. Thus, there are exciting prospects for non-invasive multi-dimensional imaging, with ample research occurring in various fields.
